# Efficacy of high-dose anakinra in refractory macrophage activation syndrome in adult-onset Still’s disease: when dosage matters in overcoming secondary therapy resistance

**DOI:** 10.1177/1759720X20974858

**Published:** 2020-11-24

**Authors:** Sofia Ajeganova, Ann De Becker, Rik Schots

**Affiliations:** Rheumatology Department, Clinical Sciences, Vrije Universiteit Brussel, Universitair Ziekenhuis, Brussels, Belgium; Department of Medicine Huddinge, Division of Gastroenterology and Rheumatology, Karolinska Institutet, Stockholm, Sweden; Department of Clinical Hematology, Universitair Ziekenhuis Brussel, Vrije Universiteit Brussel, Brussels, Belgium; Department of Clinical Hematology, Universitair Ziekenhuis Brussel, Vrije Universiteit Brussel, Brussels, Belgium

**Keywords:** Adult-onset Still’s disease, Anakinra, Macrophage activation syndrome

## Abstract

Macrophage activation syndrome (MAS) is a severe, potentially fatal complication of rheumatic diseases. This case demonstrates the significant challenges and therapeutic considerations in adult-onset Still’s disease (AOSD) complicated with MAS at initial presentation, which will be discussed. MAS in our patient was refractory to the first-line therapy with high-dose corticosteroids, early administration of anakinra at a standard dosage and subsequent add-on treatments with cyclosporine A, IVIG, etoposides and tocilizumab. At 2 months after presentation, the patient was still critically ill with clinical, laboratory and histological signs of an active uncontrolled MAS. Notably, adoption of anakinra at a high dosage finally induced remission. This case confirms that adjusted dosage of anakinra is an effective therapeutic strategy in a severe AOSD-related MAS. It is tempting to speculate that anakinra at a high dosage, if used earlier, would have significantly changed the course of the disease in our patient and could have led to earlier remission.

## Case presentation

We report here the case of a young woman in her 30s admitted to the Hematology Care Unit of the University Hospital for severe systemic inflammation. Her previous medical history was negligible, except for radiofrequency ablation for atrial arrhythmia. There was no notion of a specific triggering factor. The days before admission she had acute and high fever spikes up to 40–41°C, headache, malaise, sore throat, arthralgias and myalgias and rapid deterioration of general health. The fevers persisted to reappear, with highest temperature in the afternoon and at nights and concomitant transient maculo-papular lesions.

At admission, the clinical examination was notable for tenosynovitis of her shoulders, wrists and knees, hepatosplenomegaly and general edema. The laboratory findings were notable for a very high serum ferritin level >63,000 ng/ml, marked increased C-reactive protein (CRP) level of about 200 mg/l, severe cytolysis with lactate dehydrogenase (LDH) >5000 U/l, aspartate aminotransferase (AST) >600 U/l and alanine aminotransferase (ALT) >200 U/l, as well as thrombocytopenia of about 50,000 × 10^3^/mm^3^, fluctuating normal-to-low leucocyte count, lymphopenia, anemia, and hypoalbuminemia without proteinuria. Autoimmunity panel, such as ANA, extractable nuclear antigens (ENA), anti-dsDNA, RF, ACPA and ANCA, anti-phospholipid antibodies and microbiological analyses, including repeated hemocultures, were all negative. Findings of echographic and positron emission tomography-computed tomography (PET-CT) examinations showed hepatosplenomegaly and pleuritis, without any signs of malignancy. Histologic examination of the biopsy specimens of bone marrow showed phagocytosis of various hematopoietic cells by histiocytes.

The patient was diagnosed as having adult-onset Still’s disease (AOSD) based on three major and four minor criteria described by Yamaguchi *et al.*^1^ as well as after exclusion of concomitant infection, malignancy and autoimmune disease. According to the 2016 EULAR/ACR/PRINTO Classification Criteria,^2^ the AOSD in our patient was complicated with macrophage activation syndrome (MAS).

A day after admission, therapy with high-dose corticosteroids (CS) with methylprednisolone (MP), 1 g daily pulses for 3 days, was administered, followed by daily MP 1 mg/kg/day. However, the benefits of CS therapy were limited and only temporary, and, after a couple of days, the fevers, rash, joint and muscle pains recurred, and inflammatory signs worsened. Then, high-dose of CS pulses were given again and IL-1-blocking treatment with anakinra, at the standard dosage of 100 mg/day, was started, which induced a dramatic response with rapid disappearance of fever, rash and pains, together with an abrupt decrease in systemic inflammation and cytolysis. However, in a few days the symptoms reappeared, serum ferritin levels again increased, cytolysis and 3-lines cytopenia worsened despite ongoing treatment with anakinra and daily MP 2 mg/kg/day. The treatment was further escalated and consisted of cyclosporine A (CyA) in association with intravenous immunoglobulin, given above anakinra and high dose MP; however, no benefits were seen. Repeated bone marrow examination confirmed a phenomenon of active phagocytosis.

Because of the persistent clinical, laboratory, histology and imaging signs of an active refractory autoinflammatory disease, inflammasome activation resistant to conventional therapy and to interleukin-1 (IL-1) blocking was supposed. Considering recent reports about efficacy of interferon gamma (IFN-γ) blocking to halt severe and refractory primary haemophagocytic lymphohistiocytosis (HLH),^3^ treatment with emapalumab as an emergency investigational new drug was requested, but was unavailable for our patient with secondary HLH.

In addition to ongoing therapy with anakinra, cyclosporin A and high-dose MP, etoposide therapy was initiated, with close surveillance of possible infectious complications. Despite combined immunosuppression, the clinical status of the patient deteriorated progressively, cytopenias worsened rapidly and absolute neutrophil count was < 500; she had daily fevers, was confused and had persistent hyperferritinemia, which increased dramatically to >79,000 ng/ml. Further treatment with etoposide was considered unacceptable and, thus, was terminated after three cycles of etoposide. Repeated assessments aiming to exclude infections and malignancy were negative. Cerebrospinal fluid examination did not reveal any infection and was compatible with features of central nervous system involvement of HLH (elevation of protein and cell count). MP was increased to 2.5 mg/kg/day, and anakinra together with CyA was continued. At this point, hematopoietic stem cell transplantation was considered, and a search for suitable donors was started. Meanwhile, the clinical and neurological status of the patient improved slowly, but she had persistent fevers, severe cytolysis and hyperferritinemia.

Because of persistently active MAS resistant to IL-1 blocking, other pathogenetic pathways were supposed to maintain the inflammasome hyperactivation in our patient. Considering successful reports on efficacy of anti-IL 6 receptor antibodies in patients with refractory AOSD,^4^ IL-6 blocking with intravenous tocilizumab, at the dosage of 8 mg/kg, was administered and anakinra was stopped. In less than 3 days that followed, the patient became critically ill, with clinical symptoms and laboratory deterioration comparable with those she experienced at the time of admission.

Because only anakinra was clinically effective in our patient, even though the response was incomplete, IL-1 blocking was re-started and, as a last therapeutic resort, anakinra was given at a higher dosage, 200 mg/day (100 mg twice daily), in addition to ongoing high-dose MP and CyA. The restoration of anakinra therapy allowed clinical improvement within a few days but only partial laboratory improvement, therefore further escalation of anakinra dosage to 300 mg/day was considered. The bone marrow examination (which was repeated for the third time) and liver biopsy specimens were consistent with active phagocytosis without signs of lymphoproliferative or hepatic disease.

The following week, a persistent resolution of fever, musculoskeletal and skin complaints was achieved, and laboratory signs slowly improved. At this point, dosage of anakinra was decreased to 200 mg/day along with careful lowering of MP dosage. At 1 week after starting with tapering of immunosuppressive therapy, the patient experienced a spontaneous utero-ovarian bleeding, which was treated successfully angio-surgically. In our opinion, this event was likely attributable to the high accumulated dose of CS and persistently active MAS.

As soon as serum ferritin significantly and persistently decreased to levels below 10,000 ng/ml, anakinra was further tapered to 100 mg/day and continued thereafter. At 7 months after presentation, remission was achieved, with complete resolution of systemic and articular symptoms, normalization of serum ferritin and laboratory values, and MP was tapered and discontinued progressively. To avoid accumulation of side-effects of CyA, and, given the lack of evidence, empirically, CyA was tapered successively and finally suspended, while maintenance with oral methotrexate (MTX) was adopted. At the present time, 12 months after initial presentation, no clinical or laboratory signs of the disease have reappeared and persistent remission on treatment with anakinra and MTX has been achieved.

**Figure 1.**
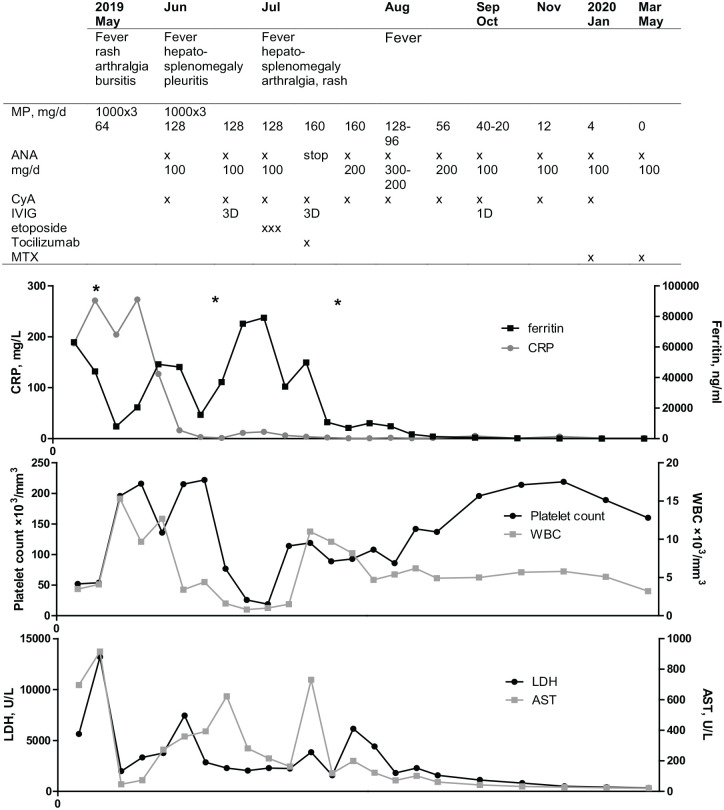
Patient’s treatments and examination results during the clinical course. After treatment with high dosage of anakinra the fevers disappeared, ferritin and all laboratory values eventually normalised. *ANA, anakinra;* AST, aspartate aminotransferase; CRP, C-reactive protein; CyA, cyclosporine A; IVIG, intravenous immunoglobulin; LDH, *lactate dehydrogenase;* MP, *methylprednisolone;* MTX, methotrexate; WBC, white blood cell count.

The course of the disease and given treatments are presented in Figure 1.

This report did not require an ethical board approval because there was no change in clinical management of the patient in routine care. The patient signed informed consent for the publication.

## Discussion

MAS is a severe, potentially fatal complication of rheumatic diseases, and shares clinical and laboratory features with primary (familial/genetic) HLH. MAS or ‘rheumatic HLH’ is classified among the secondary (acquired) forms of HLH occurring in the context of a rheumatic disease.^5^ Both the primary and secondary forms of HLH are characterized by an uncontrolled activation and proliferation of macrophages and T lymphocytes with hypersecretion of pro-inflammatory cytokines, tissue infiltration, haemophagocytosis and tissue damage. A ‘cytokine storm’ [of interleukin (IL)-1β, IL-2, IL-6, IL-18, IFN-γ, macrophage colonystimulating factor (MCSF), soluble TNF receptors, IL1R antagonist (IL1Ra), etc.] is suggested in a pathophysiological pathway of MAS, and treatments blocking various cytokines could be beneficial.^6^ The most consistent immunological abnormality described in patients with primary and secondary haemophagocytic syndrome is impairment of cellular cytotoxic function with profoundly decreased natural killer cell activity.^5^ The deficient cytotoxic function may lead to macrophage hyperactivation. Sustained macrophage activation in AOSD may lead to reactive haemophagocytic syndrome, that is, MAS, after a sudden intensification of activation, which might be related to different triggering events.

Early diagnosis and immediate therapeutic intervention are crucial for the achievement of response and resolution of MAS. There are, as of today, no available studies comparing the efficacy of treatment with IL-1 inhibitors in early *versus* late stages of AOSD. Our case is unique because it represents the use and efficacy of anakinra both in the early stage of MAS as the first-line biologic treatment and in the later stage after failure of several subsequent add-on treatments. Remarkably, at initiation of treatment with anakinra, our patient experienced rapid major improvement, whereas later the disease became resistant to several add-on therapies, which is why adoption of a high dose of anakinra was temporarily needed. The delay between institution of IL-1 blocking treatment and first major improvement was 1–2 days in the early disease, whereas the delay between adoption of high doses and significant improvement was 4 weeks in the later course of the disease. In line with reports on early IL-1 targeting in systemic juvenile idiopathic arthritis (sJIA) and AOSD,^7,8^ our case supports the notion of a ‘window of opportunity’ for first-line early anakinra treatment to prevent refractory disease. Recently, IL-1β has been identified as a crucial inflammatory mediator in the pathophysiology of AOSD. It has been stated that IL-1 inhibition represents an effective therapeutic approach in AOSD refractory to conventional treatment and/or other biologics.^7^ Consensual treatment guidelines suggest use of IL-1 inhibitors in systemic forms of AOSD as the first-line of biologic treatment as early as possible, and in AOSD-related MAS both as the first and as a subsequent line of biologic treatment.^7,9^

Anakinra, a recombinant human IL-1 receptor antagonist, blocks the biologic activity of both IL1α and IL1β by competitively inhibiting their binding to IL1R. Despite a lack of phase III trials, anakinra is widely used for the treatment of AOSD and MAS.^10–12^ Anakinra is approved at the standard dose of 1–2 mg/kg/day in children and 100 mg/day in adults with AOSD after failure of CSs, as well as for sJIA, cryopyrin-associated periodic syndromes and rheumatoid arthritis (RA). A successful clinical use of anakinra at the standard dose, in monotherapy or with concomitant DMARDs, has been described in traditional AOSD resistant to conventional therapies and, in some cases, also resistant to at least one biologic agent.^13–15^ However, this contrasts with a frequent off-label use of a higher dose anakinra in practice at the dosage up to 200 mg/day (2 mg/kg/day) among adults and 2–4 mg/kg/day among children.^16^ In this survey, the frequency of failure was higher among patients treated with anakinra at a dosage of 100 mg/day than those treated with 2 mg/kg/day.^16^ In a large Italian retrospective observational cohort, patients presented with a very aggressive systemic form of AOSD benefited from induction treatment including 200 mg/day anakinra.^9^ These findings and our observations suggest that increasing the dosage of anakinra in patients with unsatisfactory results can be a feasible therapeutic strategy for remission induction.

In both MAS and HLH, remarkably high levels of circulating cytokines have been recognised. The relative importance of these cytokines, however, is not clear. High levels of a particular cytokine that correlate strongly with disease activity do not necessarily constitute causality. By contrast, the effects of specific cytokines blocking therapies on risk of developing MAS and treatment of MAS can provide valuable clues for understanding the pathophysiology of this clinical phenomenon. Although HLH, MAS, AOSD and sJIA share pathophysiological features, it is possible that cytokine inhibitory effect of the drug at the standard dose may not be sufficient to neutralize excessive cytokine activity in HLH, MAS and severe forms of AOSD, while the ‘low’ doses could control less aggressive inflammatory condition in sJIA and traditional AOSD. Further, early therapeutic regimen based on off-label high-dose anakinra, subcutaneously or intravenously, might improve survival in patients with fulminant sHLH/MAS.^17,18^ Accordingly, off-label treatment with anakinra at a higher increasing dose, if needed, in patients with MAS has been recently recommended.^19^

Although the efficacy of anakinra has been reported in life-threatening MAS secondary both to AOSD and other rheumatic diseases including systemic lupus erythematosus and Kawasaki disease,^20–23^ data regarding the clinical course, management and outcome of MAS simultaneously presenting with AOSD is limited. This may be the result of underreporting because of overall poor prognosis in this condition. The current case adds to the observations of efficacy of high dose anakinra in a severe persistently active MAS in AOSD after an initially partial response to the standard anakinra dose. The efficacy of increasing anakinra doses to 8 mg/kg/day has been reported in a refractory AOSD complicated by MAS and myocarditis.^24^ As one would expect, and as already mentioned, refractory cases could require 100 mg anakinra several times per day; as such, in practice, it could be chosen to start at higher doses and taper the dose down when the inflammation is controlled. In most case reports describing successful use of anakinra in MAS, daily doses of up to 8–10 mg/kg/day were administered.^6^ Importantly, secondary MAS during treatment of AOSD is known to occur in patients treated with the standard anakinra dose.^6^ Analysis of the cohort of seven patients presenting simultaneously with MAS and AOSD, together with data of four literature reports, has suggested that early addition of anakinra, systemic glucocorticoids and cyclosporine as a triple regimen may improve clinical outcomes.^25^

Switching from a first to a second IL-1 inhibitor that is in both directions between anakinra (neutralizing both IL-1α and IL-1β activity) and canakinumab (a fully human monoclonal anti-IL-1β antibody designed to exclusively bind and neutralise human IL-1β, which does not bind IL-1α or IL-1Ra), could be applied, if clinically needed. In the large Italian multicentre cohort, the main reasons for switching, mostly from anakinra to canakinumab, were loss of efficacy during follow up or lack of compliance to anakinra.^16^ Surprisingly, in two phase III trials in sJIA, canakinumab at a monthly dose to 4 mg/kg was not sufficient to prevent occurrence of MAS, even when underlying sJIA was controlled.^26,27^ It is possible that this dose may not be sufficient to neutralise excessive IL-1α and IL-1β activity in MAS. Adjusting dosages of IL-1 inhibitors, by increasing the dose at each administration or decreasing the timing between injections, has proved to be a successful choice in 66.7% of patients treated with anakinra or canakinumab for different indications in the Italian cohort.^16^

In the context of a critically ill patient with a cytokine storm syndrome, it is plausible that anakinra might be preferable because of high manageability of its dose due to its short half-life (4–6 h) and large therapeutic window (1–10 mg/kg/day), compared with the much longer half-life of canakinumab (26 days), while for a maintenance regimen canakinumab might be favored. It should nevertheless be noted that the effectiveness of anakinra and canakinumab has not been compared in trials.

Notably, inhibiting IL-6 was inefficient in our patient, although IL-6 targeting with tocilizumab – a humanized anti-IL-6R antibody – is considered as an alternative to IL-1 inhibitors in patients with AOSD and refractory sJIA.^4,28,29^ Although there are similarities between the clinical features of AOSD and sJIA, there is no evidence supporting that AOSD and sJIA are the same disease; thus, different therapeutic responses to the same biologic agent could not be precluded. Indeed, levels of IFN-γ and CXCL9 have been reported to relate strictly to laboratory features of MAS, including ferritin levels, WBC, platelet counts and LDH.^30^ These correlations were not present in patients with active sJIA without MAS. These data suggest that, during MAS, but not during active sJIA, there is specific activation of the IFN-γ pathway. Available data suggest that IL6 inhibition does not provide full protection against MAS, and the excellent response of sJIA features to tocilizumab with simultaneous development of MAS features in some patients also suggests that the role of IL6 in MAS development might be limited.^31^

The relative importance of cytokines, such as IL-1, IL-6, IL-18 and IFN-γ, and the precise relationship between production of these cytokines and emergence of MAS is not clear. Differing profiles of efficacy among IL-1, IL-6 and IFN-γ inhibitors have not been established, and head-to-head comparisons are lacking. Considering available data on efficacy, IL-1 inhibition could be more suitable in systemic patients with AOSD and sJIA, while IL-6 inhibition could be more beneficial in patients with sJIA and persistently active arthritis than in patients with systemic features.^32^ A recent randomised controlled trial (RCT) in patients with AOSD did not prove the efficacy of tocilizumab over placebo according to the primary endpoint of ACR50 response at week 4, but the systemic feature score decreased more in the tocilizumab group.^33^ More severely ill patients, having thrombocytopenia and transaminase elevation (i.e. features of MAS) were not eligible for inclusion in this RCT study. However, in this trial IV tocilizumab was administered in a high dose, 8 mg/kg biweekly, and, in case of an inadequate response, additional adaptation to weekly infusion was allowed per protocol. Having in mind the importance of pathology-based treatment choice and the factor of dosing, the failure of tocilizumab in our patient could be related to withdrawal of anakinra, administration of a partial/low dose tocilizumab, or both.

This case exemplifies a failure of etoposide in MAS and could be related to the primary failure of etoposide, to an adverse effect of etoposide or to a certain genetic mutation that could have conferred further negative effect on cytolytic function and immune hyperactivation during profound cytopenia caused by etoposide in our patient. The term ‘cytokine storm’ is not unique to HLH and was first used in the context of acute graft-*versus*-host-disease, and is also used to characterize a form of systemic inflammatory response syndrome that arises as an adverse event of some chemotherapies, known as ‘HLH during chemotherapy’, and after hematopoietic stem cell transplantation.^34^ Cytokine release syndrome (CRS) may be induced by certain medications such as immune-modulating monoclonal antibody therapies and chimeric antigen receptor (CAR) T-cell therapies. This systemic inflammatory reaction shares pathophysiology and may resemble or mimic primary or secondary HLH with hyperactivation of inflammatory cells and overwhelming release of inflammatory cytokines leading to further activation of inflammatory cells, and is clinically characterized by fevers, hypotension, often marked hyperferritinemia greater than 10,000 ng/ml, liver dysfunction, cytopenia and coagulopathy.^35,36^ However, differential diagnosis is of crucial clinical relevance as management of the diagnostic entities is different. It appears that IL-6 is a key mediator of CRS, and early intervention with tocilizumab and/or corticosteroids provides good response and reversibility of severe CRS in many patients and is approved for treatment of CRS after CAR-T therapies,^36^ and could be beneficial in some patients with acute graft-*versus*-host-disease reactions,^37^ but is of limited efficacy in MAS and HLH. In our patient with MAS, subsequent treatment with tocilizumab after failure of etoposide was ineffective.

The mortality in patients with HLH is still high and new treatments are needed. The etoposide-based HLH-94 and HLH-2004 treatment protocols provide 5-year survival of 48–67% in patients with life-threatening hyperinflammatory syndrome comprising familial/genetic HLH and secondary HLH.^38^ Recently, inhibition of new cytokine targets, such as IFN-γ and also IL-18 with prohibitory effect on IFN-γ production, has emerged as a potential biological therapy of refractory HLH and MAS, and several therapeutic trials are ongoing.^39^ The efficacy of a non-etoposide regimen based on IFN-γ inhibitor emapalumab has been reported in a patient with secondary persistent HLH after failure of etoposide treatment.^40^ IFN-γ could potentially be involved also in MAS. IFN-γ levels were found to be higher in patients with active MAS at sampling compared with patients with active sJIA without MAS.^30^ The place of the new biologicals in HLH and MAS treatment strategies is yet to be established.

It is important to acknowledge that our patient had several mainstay characteristics of MAS severity at presentation, such as very high fever spikes, markedly impaired laboratory tests with remarkable hyperferritinemia and extremely high levels of LDH, pronounced thrombocytopenia, hepatosplenomegaly, CNS involvement and haemophagocytosis on bone marrow and liver biopsy.^41^ Regardless of whether severity characteristics could help in decision making regarding initial prompt adoption of aggressive treatment regimens, close monitoring of dose adaptation and therapy responses do need further evaluation.

This case raises several therapeutic difficulties in the management of AOSD-related MAS refractory to the first-line therapy. Substantial questions remain regarding therapeutic options and principles to guide the treatment and posology of this potentially life-threatening condition.

## Implications for clinical care

- Early diagnosis and immediate therapeutic intervention are crucial in the management of MAS
- Timely adoption of dosage of anakinra could overcome secondary resistance to therapy
- Recognition of features of severe MAS could help guide therapy regimes and monitoring of therapy response
